# Investigation of Chitosan Nanoparticles Loaded with Protocatechuic Acid (PCA) for the Resistance of *Pyricularia oryzae* Fungus against Rice Blast

**DOI:** 10.3390/polym11010177

**Published:** 2019-01-21

**Authors:** The Trinh Pham, Thi Hiep Nguyen, Thuan Vo Thi, Thanh-Truc Nguyen, Tien Dung Le, Do Minh Hoang Vo, Dai Hai Nguyen, Cuu Khoa Nguyen, Duy Chinh Nguyen, Trong Tuan Nguyen, Long Giang Bach

**Affiliations:** 1 Department of Science and Technology in Dak Lak province, 15A Truong Trinh, Buon Ma Thuot City 630000, Vietnam; trinhkhcn@yahoo.com; 2 International University, Vietnam National University–Ho Chi Minh City (VNU-HCMC), Thu Duc District, Ho Chi Minh City 700000, Vietnam; nthiep1981@gmail.com (T.H.N.); thanhtruc.iubme@gmail.com (T.-T.N.); 3 Institute of Applied Materials Science, Vietnam Academy of Science and Technology, 01 TL29 District 12, Ho Chi Minh City 70000, Vietnam; pompe.smile@gmail.com (T.V.T.); inpcdung@yahoo.com (T.D.L.); andy_vodo@yahoo.com (D.M.H.V.); nckhoavnn@yahoo.com (C.K.N.); 4 Graduate University of Science and Technology, Vietnam Academy of Science and Technology, Hanoi 100000, Vietnam; 5 NTT Hi-Tech Institute, Nguyen Tat Thanh University, 300A Nguyen Tat Thanh, District 4, Ho Chi Minh City 700000, Vietnam; ndchinh@ntt.edu.vn; 6 Department of Chemistry, College of Natural Sciences, Can Tho Univesity, Cantho City 900000, Vietnam; trongtuan@ctu.edu.vn; 7 Faculty of Chemical Engineering and Food Technology, Nguyen Tat Thanh University, Ho Chi Minh City 700000, Vietnam; 8 Center of Excellence for Functional Polymers and NanoEngineering, Nguyen Tat Thanh University, Ho Chi Minh City 700000, Vietnam

**Keywords:** antifungal activity, nanoparticles, chitosan, Protocatechuic Acid (PCA), *Pyricularia oryzae*, rice blast

## Abstract

In this study, chitosan nanoparticles were used as a carrier for Protocatechuic acid (PCA) to resist *Pyricularia oryzae* against rice blast. The final compound was characterized using zeta potentials for its surface electricity, Fourier transform infrared (FT-IR) analysis and transmission electron microscopy (TEM) were conducted for functional groups and for particle sizes and shape, respectively. The zeta potential results showed that loading PCA causes chitosan nanoparticle (CSNP) to decrease in surface electrons. The TEM images revealed that the particle size of chitosan (CS), although increasing in size when carrying PCA molecules, showed sufficient size for reasonable penetration into fungal cells. The FT-IR analysis showed that all functional group in CSNP carried PCA matched with previous studies. The antifungal test showed that diameters of inhibition zone of CS increases significantly after loading PCA, exhibiting the strongest antimicrobial effect on the *Pyricularia oryzae* fungus compared with weaker effects exhibited by CSNP alone or PCA. Our results suggested that CSNP loaded with PCA could be a potential compound for eradication of *Pyricularia oryzae* and that further testing on *in vitro* rice plants is recommended to reaffirm this possibility.

## 1. Introduction

The rice blast caused by fungi *Pyricularia oryzae* has a long research history in agriculture. For centuries, this disease has been observed in many Asian countries such as Japan, India, China, and Vietnam. Not until 1560, the blast was officially discovered in Italy. The disease was later found in China (1637), Japan (1760), USA (1906), and India (1913). So far, blast disease has been reported in over 85 countries around the world [1].

Food and Agriculture Organization estimated that the annual damage caused by blast is approximately 0.7 to 17.5 percent of world rice yields, equivalent to the amount of food supply for 60 million people in one year. The disease spreads in most agricultural countries and can cause epidemics in favorable conditions such as tropical environment [2]. On infested plants, lesions could be very large, up to 1.5 cm in length, and the infestation usually manifests in the form dry leaves. To treat rice blast, farmer usually use pesticides with high concentration of harmful chemicals. However, this approach is not effective and could impair the quality of the rice. Recently, nanotechnology has become the promising method for insect pest management. Nano-pesticide has been reported to provide efficient alternatives for antifungal management in agriculture without harming the environment [3,4].

Protocatechuic acid (3,4-dihydroxybenzoic acid) (PCA) is a widely-known naturally phenolic acid. PCA is similar in structure to gallic acid, caffeic acid and vanillic acid, which are typical antioxidant compounds, and is widespread in the plant kingdom [5,6,7]. PCA has been studied for its effects on human health such as antioxidant activity, anti-cancer, anti-fatigue, anti-arrhythmia, anti-inflammatory, antiviral activity, analgesics, active protection of the nerves and kidneys. There have been numerous reports of antimicrobial inhibition of PCA against *Salmonella typhimurium DT104*, *Escherichia coli O157: H7*, *Listeria monocytogenes, Staphylococcus aureus, Bacillus cereus*, and *Candida albican* [8,9,10,11]. However, the fungal pathogen resistance in plants is still an unknown possibility of PCA despite its characteristics and properties in medicine. Therefore, this research are aimed to investigate the resistance of PCA against plant pathogens, particularly the specific pathogenicity of rice blast disease.

Generally, PCA is quite stable, however, strong oxidized agents and strong bases make PCA become incompatible. Although PCA has strong antioxidant activity, the substance decomposes easily with direct contact with light and other irritants that are readily available in the environment, reducing the effectiveness of PCA activity and in turn inducing the excess use of PCA in practical applications [8,12,13,14]. Thus, solutions for this drawback need further investigation. One of the most effective and noteworthy solutions nowadays is the use of a carrier for PCA in order to ensure its activeness, saving the used substance amount and inducing slower release. To overcome such disadvantages, nanomaterials have been studied to carry PCA substrates, which could improve and further enhance the PCA activity if successful [15,16,17]. 

Chitosan is a biopolymer, a deacetylated product of chitin, and a polysaccharide found in the structural components of crustaceans such as shrimp, crabs, insects and mushroom cell walls. Chitosan has a low molecular weight as a natural, safe and ecofriendly product. Many studies have shown that this product not only stimulates plant growth but also strengthens plant resistance the infection of pathogenic microorganisms through stimulation of the plant phytoalexins [18,19]. Chitosan has been used as a raw material for the preparation of nanoparticles in recent years, because of their superior properties in nanoscale. With many features such as biocompatibility, biodegradability, membrane adhesion and non-toxicity, chitosan have become the material for many biological pharmacological applications [17,18,19,20,21,22,23,24,25,26,27,28]. In agriculture, chitosan nanoparticles (CSNP) are used as growth promoters for plants [28,29]. Experimental studies on the growth-promoting ability of chitosan nanomaterials of varying molecular weights, particularly in the form of water soluble or powdery mixes of other nutrients have studied and collected satisfactory results [28,29,30,31,32,33]. The successful synthesis of natural polymeric nanoparticle (chitosan) could contribute to the development of crop disease management and sustaining the agricultural system, especially in areas where rice crops are main income sources and traditional pesticides are prevalent.

Given the superior features and high biocompatibility described above, this study aims to overcome the disadvantages of the compound and alleviate the adverse effect of *Pyricularia oryzae* on rice by attempting the synthesis of a compound consisting of CSNP carrying PCA. Chitosan nanoparticles containing PCA were prepared by the ionic gelation method, then characterized by Transmission electron microscopy (TEM), Zeta potentials, Fourier transform infrared spectrometer (FT-IR) analysis as well as evaluated for antibacterial effects against *Pyricularia oryzae*.

## 2. Experimental

### 2.1. Materials

CS (LMW: 150 kDa, degree of deacetylation of 90%) and Sodium tripolyphosphate (TPP) were purchased from Sigma-Aldrich, St. Louis, MO, USA. PCA and *Pyricularia Oryzae* fungi were provided by Institute of Applied Materials Science, Vietnam Academy of Science and Technology, Ho Chi Minh City, Vietnam. Acid acetic 1% *v*/*v*, agar, ethanol, NaOH 5M were purchase from Xilong, China. PCA as white solid from the ethyl acetat fraction was provided by Institute of Applied Materials Science, Vietnam Academy of Science and Technology Ho Chi Minh City, Vietnam. All chemicals and solvents were of analytical grade and used as received without further purification.

### 2.2. Preparation of Chitosan Nanoparticles

The preparation of chitosan nanoparticles was achieved by ionic gelation method reviewed by a previous study with several modifications [25]. CS was dissolved in acetic acid solution (0.1%, *v*/*v*) and stirred at room temperature to gain the final concentration of 0.5% *w*/*v*. Sodium polytriphosphate (TPP) was dissolved in 10 mL deionized water to reach the concentration of 0.25% *w*/*v*. Afterwards, 2 mL of prepared TPP was added into 6 mL chitosan solution (0.5%, *w*/*v*) and stirred with the speed of 600 rpm in 30 min at room temperature. The final products were dialyzed against distilled water for 48 h to eliminate free acetic acid and residue. The supernatant was lyophilized to produce CSNP and stored at 5 °C for further analysis.

### 2.3. Preparation of Chitosan Nanoparticles Loading PCA

**Scheme 1:**
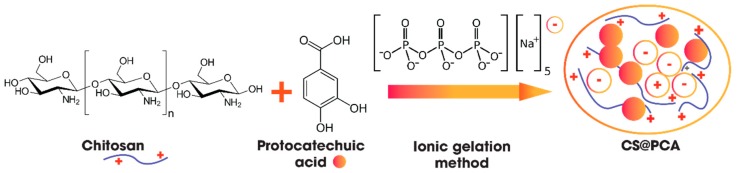
Synthesis of Chitosan loading Protocatechuic acid by ionic gelation method.

Similarly, the preparation of chitosan nanoparticles loading PCA used the same concentration of acetic acid. Adding 1 ml of PCA solution in the beginning resulted in the concentration of PCA equaling 8000 ppm. Later, TPP was added with the same parameters as the above synthesis. The final product was free-dried at −80 °C in 72 h and store at 5 °C for further analysis (See Scheme 1).

### 2.4. Characterizations

The content of PCA in CS@PCA was determined based on Folin—Ciocalteu method. After freeze-drying, an amount of CS@PCA sample was exactly weighed and then dissolved in acetic acid. Later, loading capacity (LC) of PCA and encapsulation efficiency (EE) of PCA in chitosan nanoparticles were calculated based on following equations:

$$\mathrm{LC}\left(\%\right)=\frac{\mathrm{mPCA}}{\mathrm{mCS}}\times 100$$

$$\mathrm{EE}\left(\%\right)=\frac{\mathrm{mPCA}}{\mathrm{mPCAi}}\times 100$$

where mPCA is the weight of PCA in final products, mPCAi is the initial weight of PCA, and mCS is the weight of chitosan nanoparticles.

The zeta potentials of CSNP and CS@NP were measured on a DLS (Horiba SZ—100, Horiba Scientific Ltd., Kyoto, Japan). CSNP and CS@NP were dissolved in 10 mM PBS (pH 7.4, 1 mg/mL), then sonicated for 15 min, and measured at room temperature.

The functional groups in CSNP, PCA and CS@PCA were investigated by FT-IR analysis (Nicolet Nexus 5700 FTIR, Thermo Electron Corporation, Waltham, MA, USA) in 500–4000 cm^−1^ range with KBr pellets

The sizes and morphologies of CSNP and CS@PCA solutions in diH_2_O (1 mg/mL) at 37 °C were confirmed by TEM (JEM-1400 TEM; JEOL, Tokyo, Japan). The sample solutions were dropped on a carbon-copper grid (300-mesh, Ted Pella, Inc., Redding, CA, USA) and air-dried for 10 min. Then, they were stained with 1% (*w*/*v*) negatively charged phosphotungstic acid (PTA) for 30 s before being taken to measure.

### 2.5. Agar Diffusion Test

Agar diffusion method was used to test the antifungal effect in this study. First, PDA agar plate (potato infusion at 250,000 ppm, glucose 20,000 ppm, and agar 20,000 ppm) was prepared. Next, cell suspensions from 200 µL fungal colonies were then spread directly on the agar surface. A sterilized nozzle with a diameter of 2 mm was used to create five wells on each petri disc. Then, 20 µL of each solution of CSNP, PCA and CS@PCA was put into the petri dish. The solution of CSNP, PCA and CS@PCA were prepared by dissolving in acetic acid solution of 1%. After 24 h, the diameter of inhibition zone was calculated to determine the antibacterial activity of each suspense. This experiment was repeated 3 times. The data were expressed as mean ± SD.

## 3. Results and Discussion

### 3.1. The Content of PCA in CS@PCA

**Figure 1:**
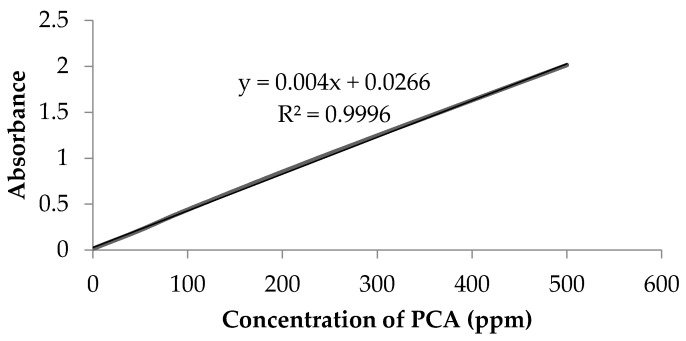
The PCA standard line.

The PCA standard line based on Folin–Ciocalteau method was established as follows (Figure 1).

$$∆OD=\frac{0.386+0.388+0.386}{3}-0.036=0.351$$

Optical density was calculated from Table 1. From the equation of PCA standard line, mPCA/2.5 mg and mPCA/sp were calculated to be 81.1 and 4499.428 µg respectively. Loading capacity and encapsulation efficiency PCA in chitosan nanoparticles were therefore as follows:

$$\mathrm{LC}\left(\%\right)=\frac{4499.428}{80000}\times 100=56.24\%$$

$$\mathrm{EE}\left(\%\right)=\frac{4499.428}{138700}\times 100=3.244\%$$

### 3.2. Zeta Potentials

**Figure 2:**
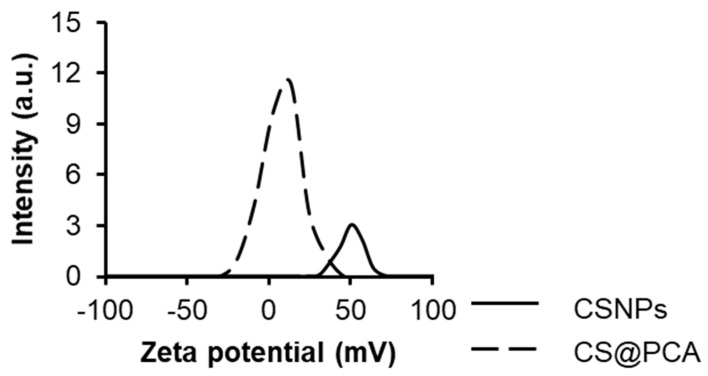
Zeta potentials of CSNPs and CS@PCA.

The surface electricity of CSNP and CS@PCA is the important factor since this is the main contractor with the fungal membrane. The abundant amount of –NH_2_ in the surface of CSNP cause this to be positively charged, enhancing the interaction with the phospholipid surface of cell membrane and assisting the release of the carrier to the fungi membrane. Figure 2 shows the zeta potentials of synthesized CSNP. The potential fluctuated from 49–52 mV. However, after loading PCA, the potentials of CSNP declined to 11 mV. This is because PCA particles reduce -NH_2_ groups on the NP surface.

### 3.3. FTIR Analysis

**Figure 3:**
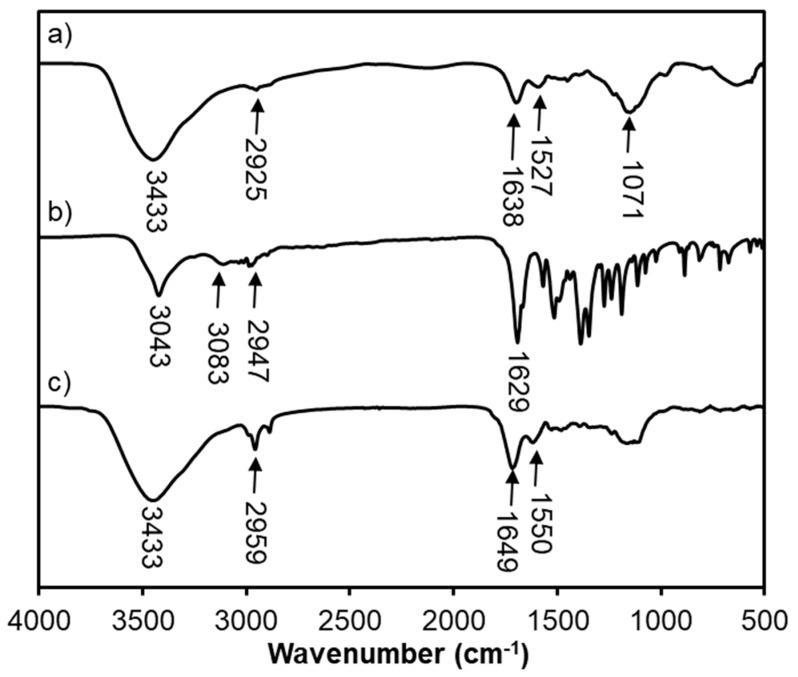
FT-IR spectra of (**a**) CSNP, (**b**) PCA and (**c**) CS@PCA.

Figure 3 demonstrates the FT-IR spectra of CSNP, PCA and CS@PCA. An FT-IR analysis of CSNP showed that the peak at 3433 cm^−1^, which was the characteristic variation of amino (–NH_2_) and hydroxyl (–OH) groups. The signals at 1071 cm^−1^ and 2925 cm^−1^ were C–O–C stretching and C–H stretching. In addition, the spectra showed the presence of signals at 1638cm^−1^ and 1527 cm^−1^, which indicated a linkage between the amino group and the phosphoric group. Thus, it can be concluded that the amine group was similarly linked to TPP in chitosan nanoparticles (TPP). The infrared spectrum of PCA showed the spike at 3403 cm^−1^ as formed by the hydroxyl group (–OH). The recorded peaks at 3083 cm^−1^ and 2947 cm^−1^ showed the expression of the C–H bond (CHO) and the peak at 1629 cm^−1^ indicated the presence of C=O group. All of the above signals matched the known molecular formula of PCA [27].

In the FT-IR spectrum of CS@PCA, the peak at 3433 cm^−1^ is evidently similar to that obtained in chitosan nanoparticles. The signal at 2925 cm^−1^ of CSNP and 2947 cm^−1^ of PCA showed the C–H bond displaced to 2959 cm^−1^, the peak at 1638 cm^−1^ of CSNP and the peak at 1629 cm^−1^ of PCA were also expressed. In addition, at 1649 cm^−1^, the signals of PCA aromatic ring were observed, but they were weaker than the original free state.

### 3.4. TEM Analysis

**Figure 4:**
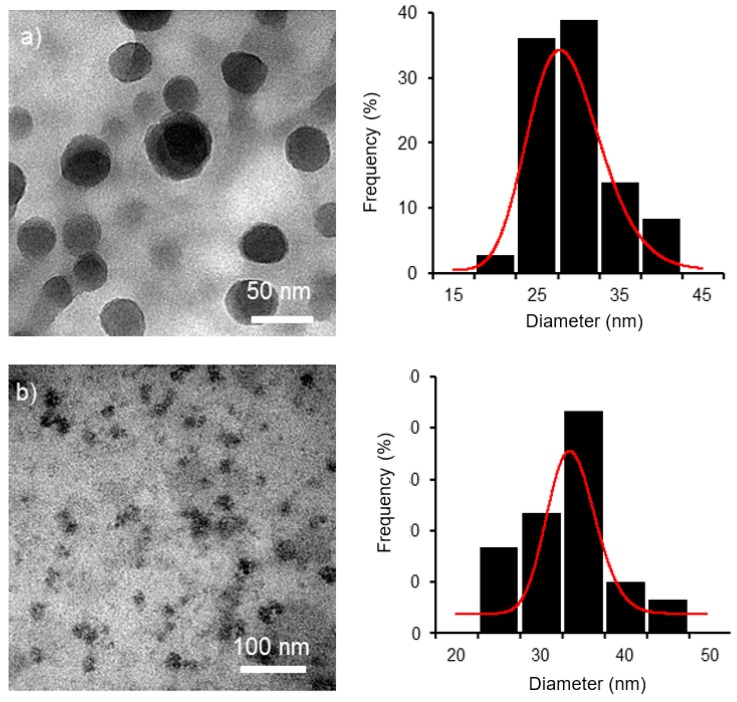
TEM images and particle size histograms of (**a**) CSNPs and (**b**) CS@PCA and their fitted by log-normal distribution function, respectively.

The TEM micrographs for CSNP and CS@PCA are shown in Figure 4. The results showed that CSNP had an average size distribution of 25–30 nm (Figure 4a) while the average nanometer size of CS@PCA was up to 30–35 nm (Figure 4b). This reveals that the particle size of CS was increased when they carried PCA molecules. This attribution of the increased size could be due to the successful bonding of PCA onto the CSNP surface. According to previous studies, particle size sufficient for good penetration into fungal cells was below 500 nm [27,28,29,30]. Thus, at this size, chitosan nanoparticles carrying PCA can fully interact on the cell membrane of the fungus, easily penetrate into the cell layer and destroy fungal cells.

### 3.5. Antifungal Activity

**Figure 5:**
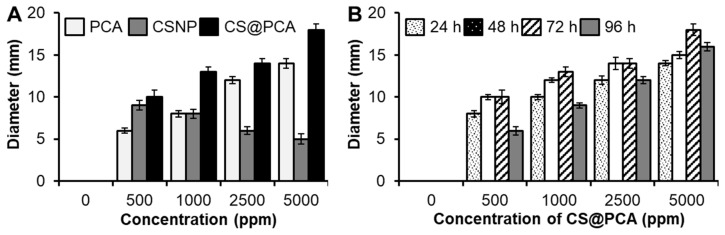
(**A**) Diameter of inhibition zone of PCA, CSNP and CS@PCA based on various concentrations of PCA as 500, 1000, 2500 and 5000 ppm and (**B**) Diameter of inhibition zone of CS@PCA after various ranges of time: 24, 48, 72 and 96 h.

**Figure 6:**
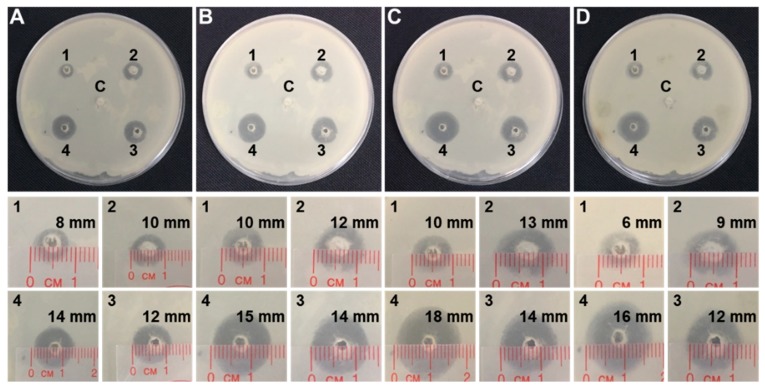
Diameter of inhibition zone of CS@PCA in 24 h (a); 48 h (b); 72 h (c) and 96 h (d) based on various concentrations of PCA as 0 ppm, 500 ppm, 1000 ppm, 2500 ppm, 5000 ppm (C = control, 1, 2, 3 and 4, respectively).

Figure 5 showed the graph of the diameter of inhibition zone of PCA, CSNP and CS@PCA with different concentrations of PCA (500, 1000, 2500 and 5000 ppm). Figure 6 focused on the diameter of inhibition zone of CS@PCA at various ranges of time (24, 48, 72 and 96 h). The results showed that both PCA, CSNP and CS@PCA exhibited anti-fungal properties. Regarding CSNP, this nanoparticle expressed the lowest antifungal activity compared with that of PCA and CS@PCA, especially after an extended period of exposure, in which its antifungal effect reduced gradually, from 9 to 5 ± 0.6 mm in diameter of inhibition zone. On the other hand, natural PCA compounds had a relatively good antifungal resistance, demonstrated by the increase of diameter in the inhibition zone (from 6 ± 0.33 to 14 ± 0,56 mm) as their concentrations spiked.

The antifungal properties of CS@PCA particles were observed for a very long time (96 h). During the experimental period, in the first 48 h, CS@PCA was observed to be resistant to the fungus, demonstrated by rising diameter up to 18 mm in the inhibition zone (Table 2. After 96 h, its antifungal activity still remained effective against the fungus despite the fact that the diameter of resistance was marginally narrowed compared to the first 48 h. From the above experiments, the antifungal activity of CS@PCA against *Pyricularia oryzae* was shown to be relatively strong compared to the effects of CSNP and PCA. The inhibitory effects of CS@PCA could be due to combinational influence of altered properties and inherent antifungal activity of chitosan. To be specific, nano-sized CS@PCA particles possessed reduced diameter and lower solubility, which facilitates the particle entry into the fungal cells and in turn fungus eradication [34]. In addition to that, lower zeta potential of synthesized CS@PCA, caused by amino groups and loaded PCA molecules, forms a positive charge and polyelectrolyte complexes by interaction with negatively charged lipopolysaccharide chains on the outer fungal membrane [35,36].

## 4. Conclusions

In this study, a novel nanopesticide fabricated from CSNP loading PCA was introduced. The zeta potential showed that PCA was loaded successfully onto the CSNP. Besides, the TEM analysis showed that the average size of CS@PCA particles, varying from 30 to 35 nm, was the optimum size of the nanoparticles for penetration into the fungal cells. Moreover, the anti-fungal antimicrobial assay of the synthesized product showed stronger anti-fungal properties of CS@PCA particles in comparison with those of the original pure PCA sample, particularly at the 5000 ppm concentration. 

## Figures and Tables

**Table 1:** Optical density of CSNPs and triplicate measurements of PCA’s weight.

Sample	Optical Density
CSNPs	0.036
PCA (1)	0.386
PCA (2)	0.388
PCA (3)	0.386

**Table 2:** Diameter of inhibition zone in case of PCA, CSNPs and CS@PCA

Concentration of PCA (ppm)	PCA (mm)	CSNPs (mm)	CS@PCA (mm)		
			24 h	48 h	96 h
500	6 ± 0.33	9 ± 0.56	8 ± 0.4	10 ± 0.3	6 ± 0.5
1000	8 ± 0.36	8 ± 0.56	10 ± 0.3	12 ± 0.3	9 ± 0.3
2500	12 ± 0.4	6 ± 0.48	12 ± 0.5	14 ± 0.7	12 ± 0.4
5000	14 ± 0.56	5 ± 0.6	14 ± 0.3	15 ± 0.4	16 ± 0.5
